# Transcriptome Analysis of Crucian Carp (*Carassius auratus*), an Important Aquaculture and Hypoxia-Tolerant Species

**DOI:** 10.1371/journal.pone.0062308

**Published:** 2013-04-22

**Authors:** Xiaolin Liao, Lei Cheng, Peng Xu, Guoqing Lu, Michael Wachholtz, Xiaowen Sun, Songlin Chen

**Affiliations:** 1 Key Laboratory of Sustainable Development of Marine Fisheries, Ministry of Agriculture, Yellow Sea Fisheries Research Institute, Chinese Academy of Fishery Sciences, Qingdao, People’s Republic of China; 2 Heilongjiang Fisheries Research Institute, Chinese Academy of Fishery Sciences, Harbin, People’s Republic of China; 3 The Center for Applied Aquatic Genomics, Chinese Academy of Fishery Sciences, Beijing, People’s Republic of China; 4 Department of Biology, University of Nebraska at Omaha, Omaha, Nebraska, United States of America; University of Lausanne, Switzerland

## Abstract

The crucian carp is an important aquaculture species and a potential model to study genome evolution and physiological adaptation. However, so far the genomics and transcriptomics data available for this species are still scarce. We performed *de novo* transcriptome sequencing of four cDNA libraries representing brain, muscle, liver and kidney tissues respectively, each with six specimens. The removal of low quality reads resulted in 2.62 million raw reads, which were assembled as 127,711 unigenes, including 84,867 isotigs and 42,844 singletons. A total of 22,273 unigenes were found with significant matches to 14,449 unique proteins. Around14,398 unigenes were assigned with at least one Gene Ontology (GO) category in 84,876 total assignments, and 6,382 unigenes were found in 237 predicted KEGG pathways. The gene expression analysis revealed more genes expressed in brain, more up-regulated genes in muscle and more down-regulated genes in liver as compared with gene expression profiles of other tissues. In addition, 23 enzymes in the glycolysis/gluconeogenesis pathway were recovered. Importantly, we identified 5,784 high-quality putative SNP and 11,295 microsatellite markers which include 5,364 microsatellites with flanking sequences ≥50 bp. This study produced the most comprehensive genomic resources that have been derived from crucian carp, including thousands of genetic markers, which will not only lay a foundation for further studies on polyploidy origin and anoxic survival but will also facilitate selective breeding of this important aquaculture species.

## Introduction

Crucian carp (*Carassius auratus*), belonging to the family Cyprinidae (Telestei), is widely distributed on the Eurasian continent [1]. Crucian carp is popular as a food fish in China and elsewhere. Due to its good taste, fast growth and suitability for mono- and polyculture in fishponds, crucian carp are one of the most intensively cultured freshwater fish in the world, with fish production reaching 2.2 million tons in 2010 according to FAO statistical data [2]. The culture of crucian carp originated a long time ago in China, and China is still the largest crucian carp-producing country in the world. Thus far, several strains of crucian carp have been developed in China based upon its unique reproductive characters (e.g., gynogenesis) [3]–[5].

Crucian carp has three different ploidy types in the wild, including diploid, triploid and tetraploid, each with a different reproductive mode [6]. Diploid crucian carp produce bisexual diploid offspring through bisexual reproduction; the tetraploid ones produce all-female offspring via gynogenesis. Interestingly, dual reproductive modes exist in triploid crucian carp. Unisexual gynogenesis by heterogenous spermatozoa activation produces all-female offspring whereas bisexual reproduction generates bisexual triploid progenies [5]–[6]. The origin of polyploidy and the maintenance of genetic diversity have not been fully understood. Crucian carp have been considered as a potential model for the study of evolutionary developmental biology (Evo-Devo) [5].

Another interesting phenomenon of crucian carp is that it can endure months of anoxia at low temperatures during the winter time. Because of this unique character, it has been considered one of the most hypoxia-tolerant fish [7], [8].The mechanisms of hypoxia-tolerance in crucian carp, although not fully understood, include large stores of glycogen, anaerobic glycolysis to maintain the adenosine triphosphate (ATP) supply and drastically decreased metabolism [8]. In addition, to avoid lactic acidosis during anaerobic glycolysis, crucian carp is able to convert lactate into ethanol as the major anaerobic end product, which is subsequently excreted through the gills [8]–[9].

These special characteristics of crucian carp suggest that it is not only a significant aquaculture species but also a potential model organism for the study of molecular mechanisms in genome evolution and physiological adaptation (e.g., anoxic survival). Indeed, very few genomic and transcriptomic resources from this species were previously available. So far only about 2,300 EST and 2,200 protein sequences are deposited in NCBI GenBank. The lack of rich genetic resources hinders not only crucian carp molecular breeding, but as well further studies on the mechanisms of specific biological processes. Fortunately, the development of high throughput next generation sequencing (NGS) technologies has provided a cost- and time-effective tool to generate both genomic and transcriptomic resources [10]–[11]. There are several reports on transcriptomic analysis in species closely related to crucian carp, using the NGS method, which have stimulated genetic and genomic studies of cyprinid species [12]–[15]. However, these studies involved transcriptomic sequencing of pooled tissues, which could not reveal tissue-specific gene expression profiles. Gene expression is context dependent, which means a gene expression profile is tissue specific. Understanding of tissue-specific gene expression can help clarify the molecular mechanisms of tissue development and function within an organism [16]–[17].In the present study, we employed the Roche 454 GS FLX platform to sequence the crucian carp transcriptome of four tissues and conducted differential gene expression analyses among tissues. This is the first report on characterizing the crucian carp transcriptome, which provides a valuable genomic resource for selective breeding and functional or evolutionary studies of genes involving hypoxia tolerance.

## Materials and Methods

### Ethics Statement

This study was approved by the Animal Care and Use Committee of the Chinese Academy of Fishery Sciences.

### Samples, Measurement of DNA Content and Tissue Collection

A total of 14 crucian carp samples (50–100 g) were collected from the fish facility in Heilongjiang Fisheries Research Institute, Chinese Academy of Fishery Sciences. To identify the ploidy types of fish samples, DNA contents were measured on a Cell Lab Quanta SC flow cytometer (Beckman Coulter, Brea, CA, USA). Red blood cells were collected from the caudal vein with syringes containing sodium heparin. The blood samples were resuspended in Nuclear Isolation Media (NIM)-DAPI staining solution (NIM-DAPI, NPE Systems, Pembroke Pines, FL, USA) for 10–15 min, and then filtered. The DNA content of color crucian carp confirmed as diploid was used as a reference standard. According to the DNA contents, we chose only diploid crucian carp for tissue collection. To obtain tissue-specific gene expression profiles and single-nucleotide polymorphism (SNP) markers, we collected four tissues (brain, muscle, liver and kidney) and each tissue mixture contained six fish samples with similar quantities. The tissue samples were stored in RNAlater reagent (Qiagen, Hilden, Germany) at −20°C before RNA isolation.

### RNA Isolation, cDNA Libraries Construction and 454 Sequencing

Total RNA from four tissues was extracted separately using TRIzol reagent (Invitrogen, Carlsbad, CA, USA). Total RNA was isolated by following the manufacturer's protocol and then treated with RNase-free DNase I (New England Biolabs) at 37°C for 30 min to remove the potential DNA. After that, RNA was suspended in RNase-free water for cDNA synthesis. cDNA was synthesized using 2 µg of total RNA using the SMART cDNA synthesis kit (Clontech Laboratories, Inc., Mountain View, CA, USA). Before cDNA synthesis, the RNA quantity and quality was checked using gel electrophoresis and Bioanalyzer (Agilent). Four cDNA libraries represented four tissues were constructed using GS FLX Titanium General Library Preparation Kit (Roche, Branford, CT, USA). To increase the probability of a rare transcript, the resulting cDNA libraries were normalized using duplex-specific nuclease (DSN) (Evrogen, Russia). Each full sequencing plate was split into two, one for each library; in total two 454 sequencing runs (plates) were conducted for the four libraries on a Roche 454 GS FLX Titanium genomic sequencer at Shanghai OE Biotech Company.

### Sequence Data Processing and *de novo* Assembly

For each raw read, low-quality bases and the sequencing adapter were trimmed using SeqClean [18] and LUCY [19]. The cleaned reads with 100 base pairs (bp) or more from four libraries were assembled using Newbler 2.5.3 with default parameters. The resulting isotig consensus sequences and remaining singletons were then considered as unigenes for the following analyses. The raw reads have been deposited to the NCBI Short Read Archive (SRA) database (accession number: SRA057034).

### Annotation

The unigenes were compared with the non-redundant (nr) protein sequences in the Swiss-Prot database using BLASTX [20] with a cutoff values of *E-values* of 1e−3 and sequence similarity ≥30%. A gene name was assigned to each unigene according to the top BLASTX hit with the highest alignment score among the blast matches. The Blast 2GO suite [21] was used to annotate unigenes to three main GO categories, biological processes, molecular functions and cellular components. The metabolic pathway analysis was performed using Kyoto Encyclopedia of Genes and Genomes (KEGG) (http://www.genome.jp/kegg/). Due to the importance of glycolysis in crucian carp oxygen tolerance, we focused on the glycolysis/gluconeogenesis pathway and analyzed the expression profile of related genes (enzymes) in the four tissues. To characterize unigenes into full-length cDNA, 5′UTR+exon(s), exon(s)+3′UTR, or exon(s), we compared crucian carp unigene sequences to all known gene sequences of zebrafish (*Danio rerio*) (downloaded from the Ensembl database) using BLASTn with a cutoff *E-value* of 1e−10.

### Comparative Transcriptomic Analysis between Crucian Carp and Other Fish Species

Now the whole sequences for eight teleost species, including zebrafish, fugu (*Takifugu rubripes*), medaka *(Oryzias latipes*), stickleback (*Gasterosteus aculeatus*), cod (*Gadus morhua*), coelacanth (*Latimeria chalumnae*), tetraodon (*Tetraodon nigroviridis*) and tilapia (*Oreochromis niloticus*), are available in the Ensembl database. We downloaded all protein sequences of the eight species from Ensembl, and then conducted a comparative transcriptomic analysis between crucian carp and these fish species using tBLASTx at *E-value* <1e−10.

### Expression Analysis

The reads from the four tissues were mapped to each correspongding isotig sequence. The expression level was calculated using the number of aligned reads to each isotig (unigene). Differentially expressed genes between tissues were identified using Fisher’s exact test with *P-value* <0.0001.

### Microsatellite and SNP Markers Discovery

The software MISA [22] (http://pgrc.ipk-gatersleben.de/misa/) was employed to discover microsatellite sequences from the unigene sequences. Five types of microsatellites were identified with criteria of di- to hexa-nucleotides motifs, and the minimum repeat unit was defined as 6 for di-, and 5 repeats for tri-, tetra-, penta- and hexa-nucleotides. The sequences composed of two or more repeat units with motifs separated by >100 bp were considered to be two or more microsatellites. Only microsatellite sequences with flanking sequences of ≥50 bp on both sides were collected for future primer designing. We used QualitySNP [23] (http://www.bioinformatics.nl/tools/snpweb/) to identify potential SNPs from isotigs containing at least 10 reads. Only those SNPs with a minor allelic frequency no less than 20% were identified. The indels were not included in SNP analysis.

## Results and Discussion

### Determination of Ploidy Types

**Figure 1:**
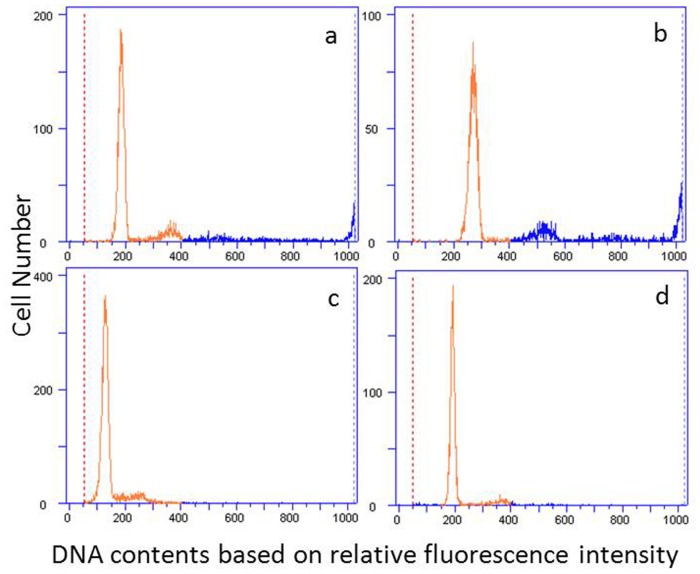
Comparison of DNA contents based on flow cytometry in crucian carp (a, b and c), with color crucian carp (d) used as a diploid reference.

Different ploidy crucian carps, cohabitating in natural waters, are difficult to distinguish based upon phenotypic characters. We thus used a time-saving and accurate flow cytometric method to identify ploidy types [6], [24].The DNA contents of 14 crucian carp samples were measured with a range from 117 to 269 based on relative fluorescence intensity, whereas the DNA content of a color crucian carp sample is 204 (**Figure 1**). According to the value of diploid color crucian carp, six crucian carp samples with DNA contents ranging from 190 to 208 were thus considered as diploids and selected for tissue collection for further total RNA isolation.

### 454 Sequencing and Assembly

**Figure 2:**
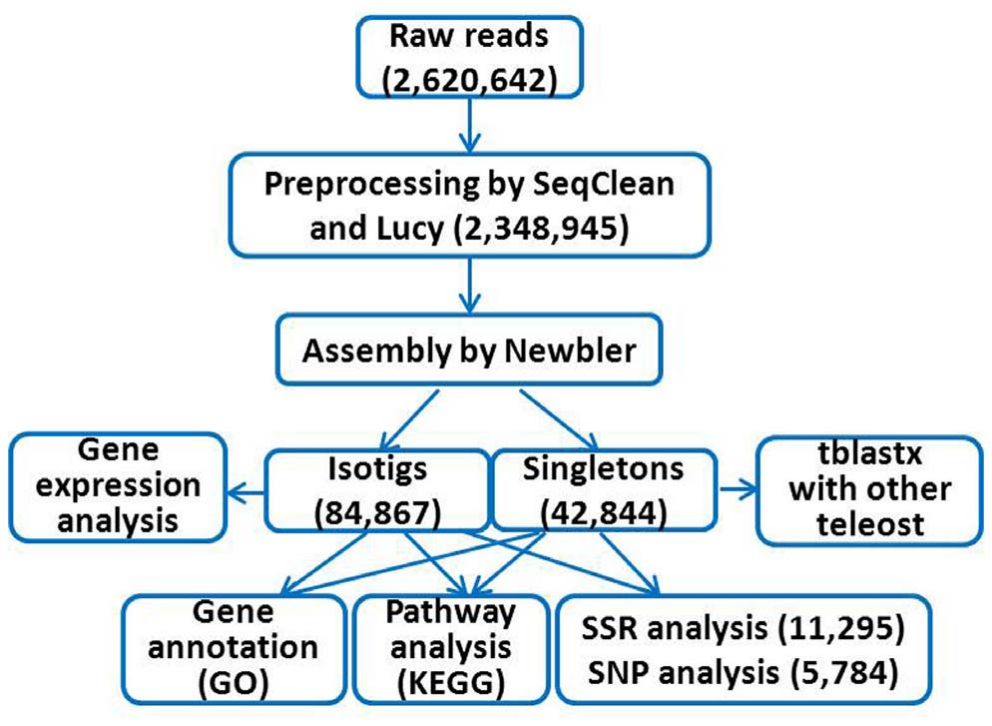
Crucian carp transcriptome assembly and analysis pipeline, with corresponding numbers (in parentheses) obtained at each step.

We constructed four cDNA libraries and subsequently obtained four sets of transcriptomic reads for crucian carp brain, muscle, liver and kidney, respectively. The analysis pipeline is showed in **Figure 2**, which begins with 454 sequencing, followed by preprocessing and assembly and ends with functional annotation and genetic markers finding.

In total, Roche 454 sequencing yielded 2,620,642 raw reads, with a total of 768,110,170 bp (768 Mbp), giving rise to 293 bp per read on average. The tissue liver was found with the largest number of raw reads (700,677), whereas the kidney tissue was found with the longest average length per read (321 bp). The tissue brain was found with the least number of raw reads (604,576) and the shortest average length per read (256 bp) (**Table 1**). After preprocessing with SeqClean and LUCY, we obtained around 2.3 million trimmed reads with ∼700 Mbp, giving rise to 293 bp per read on average. For the tissues brain, muscle, liver and kidney, 554,231, 484,255, 662,301 and 648,158 trimmed reads were generated with average length of 259, 312, 330 and 320 bp, respectively (Table 1).

**Table 1:** Statistics of crucian carp transcriptome sequences.

	Number of raw reads	Average length of raw reads(range) (bp)	Number of trimmed reads	Average length of trimmed reads(range) (bp)
Brain	604,576	256 (40–848)	554,231	259 (100–551)
Muscle	630,177	290 (40–652)	484,255	312 (100–627)
Liver	700,667	301 (40–666)	662,301	330 (100–601)
Kidney	685,222	321 (40–750)	648,158	320 (100–600)
Combined	2,620,642	293 (40–848)	2,348,945	298 (100–627)

**Figure 3:**
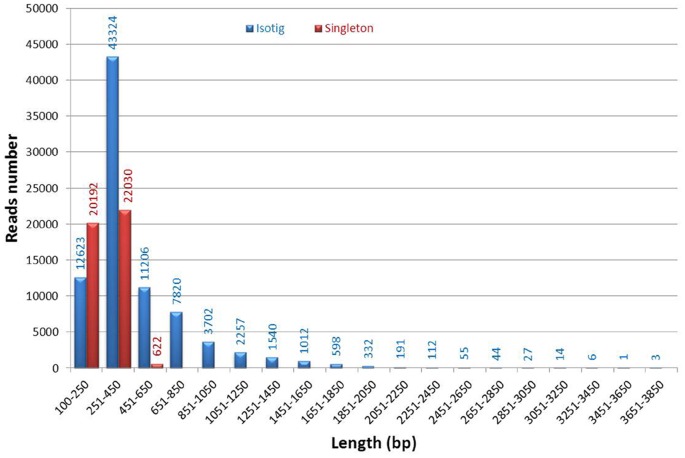
Length distribution of isotigs (blue) and singletons (red) of crucian carp transcriptome.

The assembly of cleaned reads from four combined tissues resulted in 127,711 unigene sequences, including 84,867 isotigs and 42,844 singletons (**Table 2**). The average depth of these isotigs was 27.3. The average length of isotigs was about 493 bp, and N50 was 547 bp (**Table 2**). Most (80.49%) of these isotigs distributed in the 250–1,250 bp region, but there were 3,935 isotigs (4.64%) with length over 1,250 bp. While most of the singletons distributed in the 100–450 bp region, only 622 singletons with length over 450 bp were obtained (**Figure 3**). The analysis of unigene distribution in four tissues resulted in 21,489 (50.16%), 5,745 (13.41%), 5,841 (13.63%) and 9,769 (22.80%) singletons and 67,989 (80.11%), 36,692 (43.23%), 30,710 (36.19%) and 52,132 (61.43%) isotigs in brain, muscle, liver and kidney, respectively (**Table 3**). More unigenes were found in brain and kidney than in muscle and liver. In this study, mRNA yielded from total RNA from multiple tissues of six samples using RT-PCR were sequenced, and thus the transcriptome data obtained is more comprehensive, as compared with the sequencing of a single tissue or a single sample.

**Table 2:** Statistics of crucian carp transcriptomic sequence assembly.

Number of Singletons	42,844
Number of Isotigs	84,867
Total bases of Isotigs	41,802,506
N50(bp)	547
Average length of isotigs (bp)	492.6
Average depth of isotigs	27.3

**Table 3:** Distribution of isotigs and singletons in four crucian carp tissues.

	Number of singletons	Percentage	Number of isotigs	Percentage
Brain	21,489	50.16	67,989	80.11
Muscle	5,745	13.41	36,692	43.23
Liver	5,841	13.63	30,710	36.19
Kidney	9,769	22.80	52,132	61.43

**Figure 4:**
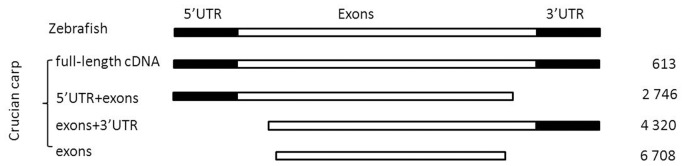
Full and partial cDNAs indentified in crucian carp as compared with zebrafish whole transcriptome.

The BLASTn search of crucian carp unigenes against all full length cDNA sequences of zebrafish downloaded from the Ensembl database showed that 12,387 unigene sequences of crucian carp were classified into four categories: 613 full-length cDNA sequences (including 140 possible ones), 2,746 exons+3′UTR sequences, 4, 320 5′UTR+exons sequences and 6,708 exons sequences (**Figure 4**). Full-length cDNA sequences have complete sequences of transcripts including coding regions (CDSs) or all exons and untranslated regions (UTRs), and thus facilitate subsequent studies on genomic structure and functional analysis [25].It is a time consuming and laborious process using the traditional Sanger sequencing method to obtain large collections of full-length cDNAs. Fortunately, the NGS method used in this study and other studies has shown to be an efficient approach to obtain a large number of full-length cDNAs [26]–[27]. In addition, over 7 000 unigenes with 5′ or 3′ UTR are also useful for obtaining full-length cDNA sequences via the primer walking method. The full-length cDNA sequences of crucian carp will be made available upon request.

### Annotation

Of 127,711 assembled unigene sequences, 22,273 (17.44% of all unigenes; including 16,331 isotigs and 5,942 singletons) had at least one significant match, corresponding to 14,449 unique protein accessions in the Swiss-Prot database (**Table S1**). A considerable percentage of sequences (82.56%) were found to have no significant match to known protein sequences. The low percentage of matched sequences might be due to too many short reads obtained from sequencing. It is known that the significance of a BLAST comparison depends in part on the length of query sequences [28].In this study, about a quarter of the unigene sequences were not very long (25.69% ≤250 bp), which might be too short to find statistically significant matches. The high number of unigenes may be attributed to the four tissues sequenced in our study. However, it is also possible that crucian carp may have more unique genes compared to other species.

**Figure 5:**
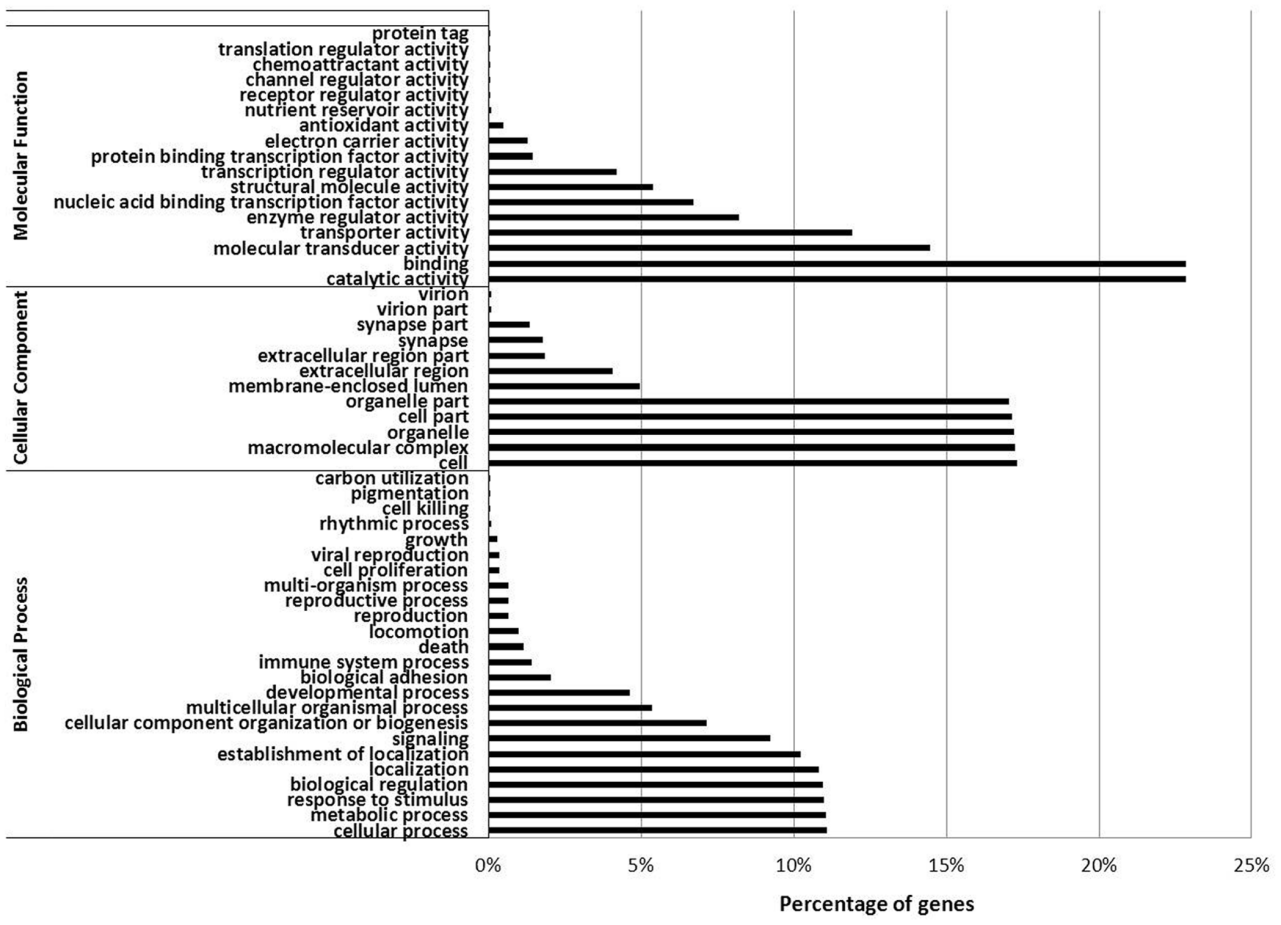
Functional classification of crucian carp unigenes based on three main Gene Ontology (GO) categories: biological process, molecular function and cellular component.

The GO analysis of the above annotated unigenes demonstrated that 14,398 (64.64%) were assigned at least one GO term, with a total of 84,876 GO assignments. For molecular functions, catalytic activity (GO: 0003824) was the most represented category, followed by binding (GO: 0005488), molecular transducer activity (GO: 0060089) and transporter activity (GO: 0005215). For cellular component, the most represented GO terms were cell (GO: 0005623), macromolecular complex (GO: 0032991), organelle (GO: 0043226), cell part (GO: 0044464) and organelle part (GO: 0044422). For biological processes, genes involved in cellular process (GO: 0009987) and metabolic process (GO: 0008152), response to stimulus (GO: 0050896), biological regulation (GO: 0065007) and localization (GO: 0051179) were highly represented (**Figure 5**).

Additionally, KEGG pathway analysis found 6,382 unigenes involved in 237 predicted KEGG metabolic pathways, which were categorized into different functional groups (**Table 4**). The number of unigenes in a predicted pathway ranged from 1 to 704 (**Table S2**). The largest functional group is organismal systems (1,853, 29.03%), which include immune system (577), nervous system (432), endocrine system (280) and digestive system (187). Unigenes grouped into metabolism, accounted for 1,680 (26.32%), including carbohydrate metabolism (346), lipid metabolism (255) and amino acid metabolism (252 sequences), etc. Cellular processes, genetic information processing and environmental information processing groups contained 1,008 (15.79%), 1 007 (15.78%) and 834 (13.04%) unigenes, respectively.

**Table 4:** KEGG biochemical mappings for crucian carp.

KEGG categories represented	Number of unigene sequences	Number of mapped KO
**Cellular Processes**	**1,008**	**743**
Cell Communication	250	179
Cell Growth and Death	294	229
Cell Motility	117	80
Transport and Catabolism	347	255
**Environmental Information Processing**	**834**	**615**
Membrane Transport	15	14
Signal Transduction	588	417
Signaling Molecules and Interaction	231	184
**Genetic Information Processing**	**1 007**	**747**
Folding, Sorting and Degradation	360	259
Replication and Repair	133	110
Transcription	142	114
Translation	372	264
**Metabolism**	**1,680**	**1,192**
Amino Acid Metabolism	252	184
Biosynthesis of Other Secondary Metabolites	22	18
Carbohydrate Metabolism	346	229
Energy Metabolism	207	149
Glycan Biosynthesis and Metabolism	155	122
Lipid Metabolism	255	166
Metabolism of Cofactors and Vitamins	103	77
Metabolism of Other Amino Acids	78	56
Metabolism of Terpenoids and Polyketides	20	16
Nucleotide Metabolism	145	107
Xenobiotics Biodegradation and Metabolism	97	68
**Organismal Systems**	**1,853**	**1,375**
Circulatory System	92	65
Development	124	90
Digestive System	187	141
Endocrine System	280	209
Environmental Adaptation	37	25
Excretory System	99	69
Immune System	577	429
Nervous System	432	326
Sensory System	25	21

### **C**omparative Transcriptomic Analysis between Crucian Carp and Other Fish Species

The unigene sequences of crucian carp transcriptome were compared with Refseq proteins of eight teleost species using tBLASTx at *E-value* <1e−10. This resulted in 10,994, 8,465, 8,546, 8,830, 8,519, 7,907, 8,398 and 9,188 unigenes with significant similarity to zebrafish, fugu, medaka, stickleback, cod, coelacanth, tetraodon and tilapia genes, respectively. Among them, there were 237, 99, 98, 106, 90, 69, 92 and 109 unigenes matched with *E-value* = 0. Crucian carp showed highest similarity to zebrafish at the gene expression level, which can be explained by crucian carp and zebrafish belonging to the same Cyprinidae family. Coelacanth is a close relative to the last common ancestor between fish and tetrapods [29]; crucian carp shared lowest similarity to coelacanth (**Table 5**). Moderate transcriptomic similarity was found between crucian carp and other teleosts.

**Table 5:** Comparative transcriptomic analyses between crucian carp and eight other teleosts.

Model species	Number of model species genes	Number of unigenes matchedto model species genes	% unigenes matched tomodel species genes	Number of unigeneswith *E-value* = 0	Average*E-value*
zebrafish	26,212	10,994	41.9	237	1.38e−12
tilapia	21,437	9,188	42.9	109	1.69e−12
stickleback	20,787	8,830	43.4	106	1.62e−12
medaka	19,686	8,546	43.4	98	1.55e−12
cod	20,095	8,519	42.4	90	1.84e−12
fugu	18,523	8,465	45.7	99	1.63e−12
tetraodon	19,602	8,398	42.8	92	1.71e−12
Coelacanth	19,567	7,907	40.4	69	1.84e−12

### Differential Analysis of Gene Expression Profiles between Tissues

**Figure 6:**
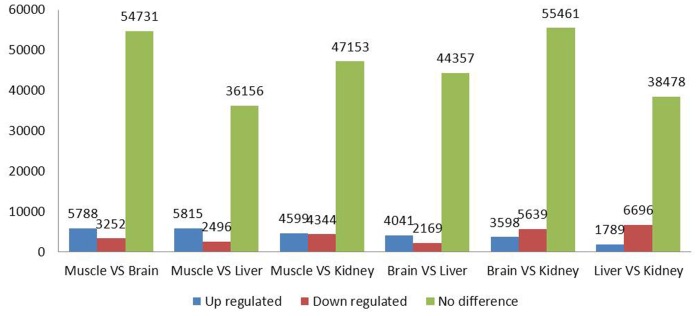
Differential gene expression analysis between four different tissues of crucian carp.

Differential expression analysis of unigenes (isotigs) demonstrated that most genes were found to have no significant difference between tissues, with the number of unigenes ranging from 36,156 to 55,461 (**Figure 6**). In contrast, the number of up- and down-regulated genes ranged from 6,210 (brain vs liver) to 9,237 (brain vs kidney). The whole transcriptomic analysis showed more genes up-regulated in muscle and more genes down-regulated in liver compared with genes expressed in other tissues. This can be interpreted by the observation that muscle and liver are crucial organs respectively for growth and environmental adaptation. In addition, the differential genes found in our study can be used as potential molecular markers for crucian carp selection and breeding to improve aquaculture production. However, further confirmation of the role of these genes is required using other more sensitive techniques such as real time PCR and with more focused pathway analysis (see the next section). The information on unigenes (isotigs) and the corresponding number of reads are detailed in **Table S3**.

### Glycolysis/Gluconeogenesis Pathway Analysis

**Figure 7:**
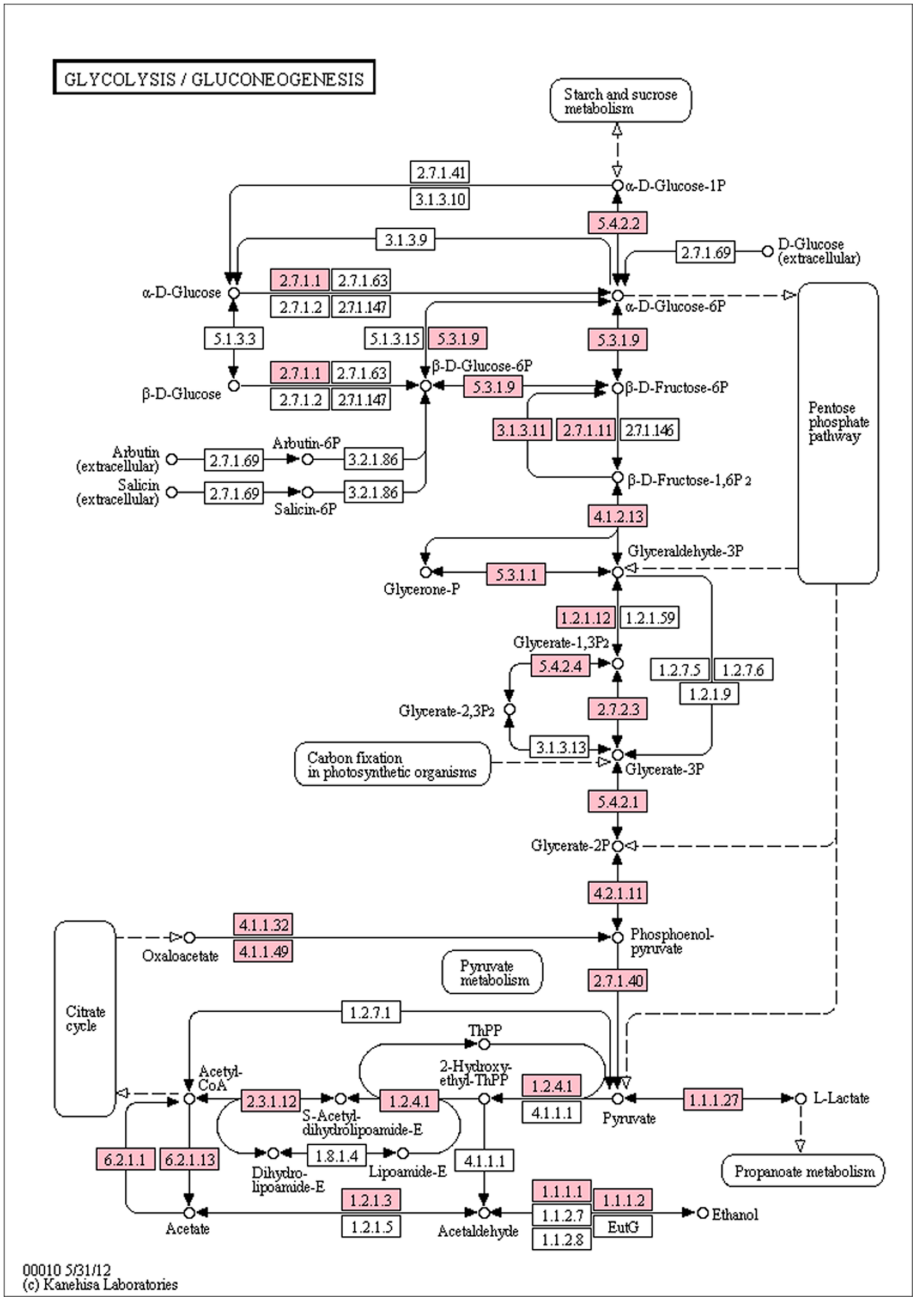
Glycolysis/gluconeogenesis KEGG map populated with transcripts coding for corresponding enzymes. The enzymes identified are shown in red boxes and their abundances of transcripts in different tissues are shown in **Table 6** **.**

Crucian carp begin to store large amount of glycogen in liver and muscle in autumn. The stored glycogen can be consumed through anaerobic glycolysis for months to keep ATP levels up, which allows crucian carp to survive during hypoxia conditions in winter [30].To gain further insights into the molecular mechanism of hypoxia tolerence in crucian carp, KEGG glycolysis/gluconeogenesis pathway (MAP00010) was populated with unigenes identified in this study. With Blast2GO, 43 unigenes that mapped to 20 enzyme genes were found to be associated with this pathway. To explore whether more unigenes can be found in crucian carp isotigs or singletons, we downloaded from Ensembl database all the transcripts for the zebrafish genes involved in the glycolysis/gluconeogenesis pathway and queried them against the crucian carp transcriptome using Blastn with *E-value* <1e−10. After manual curation of the matches, we identified 168 unigenes related to this pathway, which code for 23 enzymes (pink boxes, **Figure 7**). Three additional enzymes, phosphoenolpyruvate carboxykinase 2 [EC: 4.1.1.49], acyl-CoA synthetase [EC: 6.2.1.13] and bisphosphoglycerate mutase [EC: 5.4.2.4]) were identified only by the above method. The expression of the above enzymes varies in different tissues (**Table 6**). Highly expressed enzymes were phosphoglycerate kinase [EC: 2.7.2.3], phosphoglycerate mutase [EC: 5.4.2.1], phosphoenolpyruvate carboxykinase 2 [EC: 4.1.1.49], while enzymes such as acyl-CoA synthetase [EC: 6.2.1.13] and bisphosphoglycerate mutase [EC: 5.4.2.4] were least expressed (**Table 6**). As shown in Table 7, more transcripts have been found in muscle, especially for phosphoenolpyruvate carboxykinase 1 [EC: 4.1.1.32], phosphoenolpyruvate carboxykinase 2 [EC: 4.1.1.49] and pyruvate dehydrogenase [EC: 1.2.4.1]. A previous study conducted in three fish species suggested that the glycolytic enzymes were found in all tissues, but more in muscle than in other tissues [31], which is in agreement with the findings in the present study. In comparison with other anoxic animals such as frog (*Rana pipiens*), turtle (*Chrysemys picta*) and shark (*Hemiscyllium ocellatum*), crucian carp has similar responses to the absence of oxygen, such as profound metabolic suppression and tolerance of ionic disturbance, but can maintain active and responsive functions in oxygen absence [10], [32]–[33]. Additionally, to avoid lactic self-poisoning during glycolysis, crucian carp has a specific ability to produce ethanol and release it into water through gills [9].Hypoxia could be an important evolutionary driving force resulting in both convergent and divergent physiological strategies for hypoxic survival [33]. In this study we investigated the genes involved in glycolysis and gluconeogenesis in crucian carp, comparative analysis of these genes among hypoxia tolerant animals, along with the study of other important genes such as hypoxia-inducible transcription factor (HIF) [33]–[34] may offer clues for the molecular adaptation involved in hypoxia tolerance.

**Table 6:** The abundance of transcripts involved in glycolysis and gluconeogenesis in four tissues.

Enzyme code	Enzyme Name	Brain	Muscle	Liver	Kidney	Total
EC:2.7.2.3	phosphoglycerate kinase	193	113	175	184	665
EC:5.4.2.1	phosphoglycerate mutase	21	163	33	161	378
EC:4.1.1.49	phosphoenolpyruvate carboxykinase 2	0	339	6	23	368
EC:4.1.2.13	fructose-bisphosphate aldolase	39	158	29	133	359
EC:4.1.1.32	phosphoenolpyruvate carboxykinase 1	0	353	0	5	358
EC:2.7.1.1	hexokinase	51	23	64	132	270
EC:4.2.1.11	enolase	61	12	121	58	252
EC:1.1.1.2	aldehyde reductase	4	100	23	112	239
EC:1.2.1.3	aldehyde dehydrogenase	16	85	3	133	237
EC:1.2.4.1	pyruvate dehydrogenase	1	227	2	5	235
EC:5.3.1.9	phosphoglucose isomerase	16	57	4	154	231
EC:2.7.1.11	phosphofructokinase	11	45	31	82	169
EC:1.1.1.27	L-lactate dehydrogenase	20	31	74	4	129
EC:3.1.3.11	fructose 1,6-bisphosphatase	2	70	5	50	127
EC:6.2.1.1	acyl-CoA synthetase short-chain family member 2	23	50	5	42	120
EC:1.2.1.12	glyceraldehyde 3-phosphate dehydrogenase	13	62	11	33	119
EC:1.1.1.1	alcohol dehydrogenase	0	57	1	49	107
EC:5.3.1.1	triosephosphate isomerase	45	13	7	8	73
EC:2.7.1.40	pyruvate kinase	6	28	1	10	45
EC:2.3.1.12	dihydrolipoamide S-acetyltransferase	1	23	0	8	32
EC:5.4.2.2	phosphoglucomutase	0	10	6	1	17
EC:6.2.1.13	acyl-CoA synthetase	1	11	0	0	12
EC:5.4.2.4	bisphosphoglycerate mutase	1	0	0	0	1

**Table 7:** Statistics of microsatellites identified from crucian carp transcriptome.

Microsatellites identified	11,295
Di-nucleotide repeats	8,406
Tri-nucleotide repeats	2,032
Tetra-nucleotide repeats	760
Penta-nucleotide repeats	87
Hexa-nucleotide repeats	10
Number of isotigs and singletons containing microsatellites	10,203
Number of microsatellites with sufficient flanking sequence (≥50 bp) for PCR primer design	5,364

### Transcriptome-derived Molecular Markers Development

**Figure 8:**
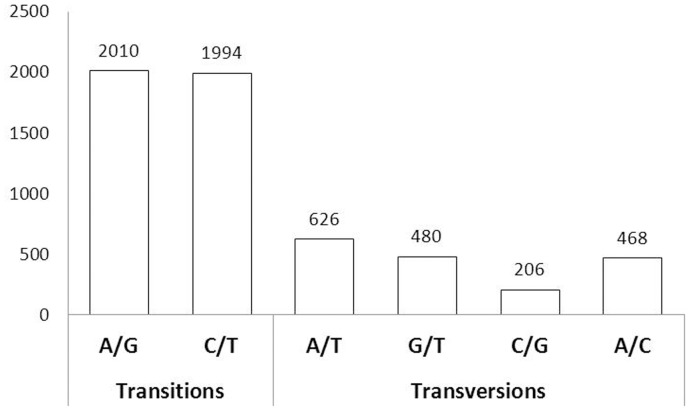
Classification of single nucleotide polymorphisms (SNPs) identified in the crucian carp transcriptome.

Crucian carp is an important aquaculture fish. Molecular breeding, such as marker-assisted selection (MAS), is expected to have great potential to increase aquaculture production [35]. In addition, molecular markers are essential to the studies of population genetics, biogeography, and evolution [3], [4], [24], [36]. However, only a few microsatellite markers (or Simple Sequence Repeat, SSR) are available in crucian carp [37]–[39].The transcriptomic sequences obtained through NGS provided an excellent source for mining and development of transcriptome-derived (or gene-associated) molecular markers [10], [13]–[15], [28]. In total, 11,295 microsatellites were identified from 10,203 unigene sequences (**Table 7**). The most frequent repeat motif was di-nucleotide repeat (8,406) that accounted for 74.44% of all microsatellites, followed by tri- (2,032, 17.99%), tetra- (760, 6.73%), penta- (87, 0.77%), and hexa-nucleotide repeats (10, 0.09%). Considering future use of these markers, 5,364 unique sequences with microsatellites were found to have sufficient flanking sequences (≥50 bp) on both sides for primer design (**Table 7**). Under stringent measures using QualitySNP and without consideration of indels, 5,784 putative high-quality SNPs were identified, including 4,004 transitions and 1 780 transversions (**Figure 8**). According to the stringent quality assessment measures of SNPs proposed by Wang et al. [40], SNPs harbored in contigs containing at least four ESTs and a minor allele sequence appearing at least twice were reliable for genotyping. In the present study, all the predicted SNPs were identified from isotigs covered by at least 10 reads with a minor allelic frequency no less than 20%, suggesting the SNPs identified in the present study were more likely to be authentic. These putative SNPs can thus be used as priority candidates for further genetic-environmental or genotype-phenotype association studies in crucian carp.

### Conclusions

We performed *de novo* transcriptome sequencing of crucian carp brain, muscle, liver and kidney tissues (each with six specimens), obtained 127,711 unigene sequences, and analyzed their Gene Ontology categories and pathways. The differential gene expression analyses revealed more genes expressed in brain, up-regulated in muscle and down-regulated in liver as compared with genes expressed in other tissues. Importantly, we identified 11,295 microsatellite and 5,784 SNP markers for crucian carp molecular selection and breeding. The transcriptome of crucian carp obtained in this study is a valuable resource for aquaculture and basic research such as genome evolution (e.g., polyploidization) and physiological adaptation (e.g., hypoxia tolerance).

## Supporting Information

Table S1 Annotation information of crucian carp transcriptome, including unigene name, length, database sequence length, unigene alignment, database sequence alignment, annotation, E-value and identity.
(XLS)

Table S2 Detailed information of KEGG pathway analysis.
(XLS)

Table S3 Gene differential expression analysis: reads number of isotigs in four tissues and Fisher’s P-value.
(RAR)
